# Genital microbial profile of repeat breeding in a Kangal sheep flock: a survey of ewes and rams under natural mating

**DOI:** 10.5194/aab-69-619-2026

**Published:** 2026-11-05

**Authors:** Abdurrahman Takcı, Mahmut Niyazi Moğulkoç, Mehmet Buğra Kıvrak, Recep Kalın, Mehmet Akif Demirel, Gamze Sönmez Ünal

**Affiliations:** 1 Department of Obstetrics and Gynecology, Sivas Cumhuriyet University Faculty of Veterinary Medicine, Sivas 58000, Türkiye; 2 Department of Microbiology, Sivas Cumhuriyet University Faculty of Veterinary Medicine, Sivas 58000, Türkiye; 3 Clinic Obstetrics and Gynecology, Sivas Numune Hospital, Sivas 58000, Türkiye

## Abstract

Infertility in sheep remains a significant concern in modern livestock production, with non-pregnancy rates during the breeding season increasing considerably in recent decades. The present study was conducted in a Kangal sheep flock with a documented history of reproductive failure, characterised by consecutive estrous cycles without conception.

A total of 905 multiparous Kangal ewes (3–6 years old) and 44 Kangal rams (4–6 years old), all with documented fertility records, were enrolled at the beginning of the breeding season. Before mating, all ewes underwent transrectal ultrasonographic examination and evaluation of vaginal discharge. Following this screening, 880 ewes with no ultrasonographic abnormalities and normal vaginal discharge were monitored throughout the breeding season. Of these, 171 ewes that failed to conceive after the first mating underwent a second mating cycle, resulting in 50 additional pregnancies. The remaining 121 non-pregnant ewes underwent a third mating, resulting in 36 additional pregnancies. Blood samples were subsequently collected from the 85 ewes that remained non-pregnant after three consecutive mating attempts. Of these, 21 tested seropositive for *Coxiella burnetii* and border disease virus (BDV) and were excluded from the study. Vaginal swabs were collected from 25 of the remaining 64 ewes that tested negative for *Brucella* spp., *C. burnetii*, and BDV. These animals were classified as repeat breeders or clinically affected animals (Group 2). An equal number of ewes ( n=25 ) that tested negative for these pathogens and conceived after a single mating cycle were assigned to the healthy control group (Group 1). Preputial swabs were also collected from 15 breeding rams to evaluate the potential for venereal transmission.

All swab samples were subjected to microbiological culture and polymerase chain reaction (PCR) analysis following DNA extraction. The targeted pathogens included *Brucella* spp., *Mycoplasma* spp., *Chlamydophila* spp., *Campylobacter* spp., *Coxiella burnetii*, *Salmonella* Abortusovis, *Trueperella pyogenes*, and *Klebsiella pneumoniae*. Although *Mycoplasma* spp., *T. pyogenes*, and *Campylobacter* spp. were detected in swab samples from both groups, the prevalence of *Mycoplasma* spp. ( $p_{\mathrm{adj}}<0.001$ ) and *Mycoplasma bovigenitalium* ( padj=0.05 ) was significantly higher in Group 2 than in Group 1. The increased detection of these microorganisms in reproductively compromised ewes suggests a potential role in the aetiology of ovine infertility, either through direct pathogenic effects or by contributing to reproductive tract dysbiosis. However, further controlled studies are warranted to establish a definitive causal relationship between these microorganisms and reproductive failure in sheep.

## 1 Introduction

To maximise meat and milk production in sheep, reproductive performance must be effectively maintained through appropriate reproductive management (Abecia et al., 2012). Compared with cattle, sheep are exposed to fewer factors adversely affecting fertility because they experience a prolonged period of seasonal anoestrus between lambing and the subsequent breeding season. The average conception rate in ewes during the breeding season is approximately 90 %, whereas the target pregnancy rate is 95 %. Similarly, although the average lambing rate is around 80 %, the target is 95 % (Kennedy, 2012; Ratanapob et al., 2020). Because lambing rate is a key determinant of flock profitability, even a small increase can substantially improve economic returns. Achieving the target lambing rate is therefore essential for the economic sustainability of sheep enterprises (Krupová et al., 2014). Despite these reproductive targets, several factors compromise pregnancy maintenance and the production of healthy offspring. One of the most important is embryonic loss, defined as the termination of pregnancy for any reason by day 34 after mating. Embryonic loss is classified as early (days 0–12) or late (days 13–33). It is commonly associated with impaired maintenance of progesterone concentrations resulting from either infectious or non-infectious causes (Givens and Marley, 2008). Infectious causes, in particular, tend to occur at the flock level rather than as isolated individual cases. Consequently, outbreaks of reproductive disorders can develop rapidly and lead to substantial economic losses and reduced flock productivity (Menzies, 2007; Mobini, 2007). Failure to establish pregnancy despite regular estrous cycles and repeated mating may indicate embryonic loss before day 12 of gestation. This is generally associated with an inadequate uterine environment for embryo implantation; and when it affects more than 5 % of ewes within a flock, it represents a significant reproductive management problem. In contrast, failure to conceive accompanied by a prolonged return to oestrus after mating is considered indicative of embryonic loss occurring after day 12 of gestation (Menzies, 2011).

Failure to achieve pregnancy after three or more consecutive matings in farm animals is suggestive of the repeat breeder phenomenon. Despite considerable advances in reproductive management, repeat breeding remains a major challenge to achieving optimal reproductive performance in livestock. Although this condition has not been fully investigated in sheep, it is defined in dairy and beef cattle as the failure to achieve pregnancy after three consecutive regular estrous cycles and inseminations using fertile semen or mating with a fertile male, in the absence of anatomical abnormalities or clinically apparent infectious disease (Pérez-Marín and Quintela, 2023). In sheep, both specific and non-specific pathogens may increase the return-to-oestrus rate by disrupting the embryonic environment. Specific pathogens – including bacteria, viruses, protozoa, and mycoplasmas – are believed to reach the uterus via hematogenous or ascending vaginal routes. In contrast, non-specific pathogens, predominantly bacteria, are thought to enter the uterus through ascending infection. These pathogens may induce embryonic loss through endometritis or direct cytolytic effects without producing overt clinical signs. Less frequently, they may contribute to embryonic loss through systemic effects in the ewe. Nevertheless, the microorganisms responsible for infertility in sheep have not been fully characterised, largely because infertility in this species has received relatively limited attention (Shah, 2019). Accordingly, rather than screening for bacteria commonly isolated from the healthy genital tract (Cassas et al., 2024), the present study focused on pathogens known to be associated with uterine diseases in ruminants, including metritis and endometritis, namely *Brucella* spp., *Mycoplasma* spp., *Chlamydophila* spp., *Campylobacter* spp., *Coxiella burnetii*, *Salmonella* Abortusovis, *Trueperella pyogenes*, and *Klebsiella pneumoniae* (Nicholas et al., 2008; Reinoso-Peláez et al., 2025; Rzewuska et al., 2019).

Based on this background, the present study was conducted to investigate the causes of the increasing incidence of infertility in sheep. Estrous cycles were monitored, and mating records were maintained throughout a single breeding season in a sheep flock with a history of infertility. Ewes that failed to conceive and the rams used for natural mating were subsequently sampled and examined for the presence of potential causative pathogens.

## 2 Material and methods

### 2.1 Location

The study was conducted on a sheep farm located in Gökçeyazı Village, Gürün District, Sivas Province, Türkiye (latitude: 38.871021° N; longitude: 37.306164° E; altitude: 1790 m). The farm is situated in a mountainous steppe region characterised by extensive natural pastureland.

### 2.2 Animals and study protocol

To comprehensively investigate the causes of infertility, a sheep flock with a history of reproductive failure in previous years was selected for the study. At the beginning of the breeding season, a total of 905 multiparous Kangal ewes (aged 3–6 years, weighing 58–66 kg, with a documented history of fertility) and 44 Kangal rams (aged 4–6 years, weighing 112–128 kg, with proven fertility) were included. Prior to mating, all ewes underwent ultrasonographic examination and evaluation of vaginal discharge. Discharge characteristics were assessed. Ewes with purulent, mucopurulent, or serosanguineous discharge were excluded from the study. Ewes with odourless discharge free of particulate content were included in the study. Following this screening, 880 ewes with normal vaginal discharge and no pathological abnormalities of the ovaries or uterus were monitored throughout the breeding season. Matings were recorded. Following each mating, pregnancy examinations were performed on the ewes via transrectal ultrasonography at intervals of 16–21 d. After the first mating, 171 ewes that failed to conceive underwent a second mating, resulting in 50 additional pregnancies. The remaining 121 non-pregnant ewes were mated for a third time, resulting in 36 further pregnancies. Blood samples were collected from the 85 ewes that remained non-pregnant after three consecutive matings. Of these, 21 tested positive for *Coxiella burnetii* or border disease virus (BDV) and were sent to slaughter. Vaginal swab samples were randomly collected from 25 of the remaining 64 ewes that tested negative for *Brucella* spp., *Coxiella burnetii*, and BDV. These animals were classified as repeat breeders and designated as Group 2. In addition, 25 multiparous ewes of similar age and body weight that conceived after a single estrous cycle were classified as healthy and assigned to the healthy control group (Group 1). These animals were randomly selected from the fertile ewes in the flock that conceived following a single estrous cycle. Preputial swab samples were collected from 15 of the 44 breeding rams used during the breeding season. The study population comprised 25 fertile control ewes, 25 repeat breeder ewes, and 15 breeding rams selected from the same flock according to the sampling procedures described above.

### 2.3 Samples

Vaginal swab samples were collected from 25 of the 64 ewes that tested negative for *Coxiella burnetii*, *Brucella* spp., and BDV. In addition, preputial swab samples were collected from 15 breeding rams. Duplicate swab samples were transported to the microbiology laboratory at approximately 4 °C immediately after collection.

### 2.4 Culture

Swab samples were serially diluted in *Mycoplasma* broth (PPLO broth without crystal violet; Becton, Dickinson and Company (BD), Franklin Lakes, NJ, USA) and incubated under microaerophilic conditions with 10 % CO_2_ for 1 week. The broth cultures were examined daily for turbidity before being subcultured onto *Mycoplasma* agar (BD, USA), following general culture procedures described by Nicholas and Baker (1998) and WOAH (2018).

### 2.5 DNA isolation

Following inoculation of each swab sample into a microcentrifuge tube containing 1 mL of sterile saline solution, DNA was extracted using a phenol:chloroform:isoamylalcohol ( 25:24:1 ; Fluka) extraction procedure, based on the general DNA extraction methodology described by Bashiruddin (1998). The extracted DNA was subsequently diluted with distilled water and stored at - 20 °C until further analysis.

### 2.6 PCR analysis

To identify the causative agents associated with repeat breeder cases, PCR analyses were performed using the primer sets listed in Table 1. Briefly, 50 µ L PCR reaction mixtures were prepared containing 5 µ L of 10× PCR buffer (500 mM KCl, 100 mM Tris-HCl (pH 9.1), and 0.1 % Triton X-100; Vivantis, ViBuffer A), 5 µ L of 25 mM MgCl_2_ (Vivantis), 250 µ M of each dNTP (Vivantis, NP2406, 100 mM), 20 pmol of each primer pair (Macrogen, South Korea), 1.25 U of Taq DNA polymerase (Vivantis PL1202, 500 U), and 5 µ L (25 ng) of template DNA. DNA amplification was performed using a T100 Thermal Cycler (Bio-Rad Laboratories, USA), following the amplification conditions reported in the corresponding methodological references listed in Table 1.

PCR products were separated by electrophoresis on a 1.5 % ( w/v ) agarose gel, stained with ethidium bromide for 30 min, and visualised using a Quantum ST4 gel documentation system (Vilber Lourmat, France), following the general procedure described by Lee et al. (2012).

**Table 1:** List of primers for PCR of swab samples obtained from sheep and rams.

Agent	Primer sequence	Amplicon	Reference
*Brucella* spp.	TCGAGCGCCCGCAAGGGG	905 bp	Romero et al. (1995)
	AACCATAGTGTCTCCACTAA		
*Mycoplasma* spp.	GGCGAATGGGTGAGTAACACG	464 bp	Wong-Lee and Lovett (1993)
	CGGATAACGCTTGCGACCTATG		
*M. canis*	TGATGATTAGCTGATAGTAGAACT	434 bp	Nicholas et al. (2008)
	GATTTGCTTGACGTCGCCGTT		
*M. alkalescens*	GCTGTTATAGGGAAAGAAAACT	704 bp	Kobayashi et al. (1998)
	AGAGTCCTCGACATGACTCG		
*M. arginini*	GCATGGAATCGCATGATTCCT	545 bp	Timenetsky et al. (2006)
	GGTGTTCTTCCTTATATCTACGC		
*M. bovirhinis*	GCTGATAGAGAGGTCTATCG	316 bp	Kobayashi et al. (1998)
	ATTACTCGGGCAGTCTCC		
*M. bovigenitalium*	CGTAGATGCCGCATGGCATTTACGG	312 bp	Kobayashi et al. (1998)
	CATTCAATATAGTGGCATTTCCTAC		
*M. agalactiae*	CTCAAAAATACATCAACAAGC	1627 bp	Subramaniam et al. (1998)
	CTTCAACTGATGCATCATAA		
*M. putrefaciens*	ACTGAGATACGGCCCAGAC	389 bp	Le Grand et al. (2004)
	ACTTGAGTCGCGGCGTCGA		
*M. ovipneumoniae*	TGAACGGAATATGTTAGCTT	361 bp	McAuliffe et al. (2003)
	GACTTCATCCTGCACTCTGT		
*Mycoplasma capricolum* subsp. *capripneumoniae* (Mccp)	ATCATTTTTAATCCCTTCAAG	318 bp	Woubit et al. (2004)
	TACTATGAGTAATTATAATATATGCAA		
*M. dispar*	TTAAAGCTCCACCAAAAA	433 bp	Marques et al. (2007)
	GTATCTAAAGCGGACTAAA		
*M. mycoides* cluster	GAAACGAAAGATAATACCGCATGTAG	785 bp	Le Grand et al. (2004)
	CCACTTGTGCGGGTCCCCGTC		
*C. burnetii*	TATGTATCCACCGTAGCCAGTC	687 bp	Berri et al. (2000)
	CCCAACAACACCTCCTTATTC		
*Chlamydophila* spp.	CTGAAACCAGTAGCTTATAAGCGGT	110 bp	Kemmerling et al. (2009)
	ACCTCGCCGTTTAACTTAACTCC		
*Cp. abortus*	CTCACCATTGTCTCAGGTGGA	821 bp	Berri et al. (2009)
	ACCGTAATGGGTAGGAGGGGT		
*Cp. pecorum*	TTCGACTTCGCTTCTTACGC	526 bp	Berri et al. (2009)
	TGAAGACCGAGCAAACCACC		
*Campylobacter* spp.	GGAGGATGACACTTTTCGGAGCG	840 bp	Vanniasinkam et al. (1999)
	TCGCGGTATTGCGTCTCATTGTATATGC		
*C. fetus* subsp. *fetus*	GGTAGCCGCAGCTGCTAAGAT	964 bp	Hum et al. (1997)
	TAGCTACAATAACGACAACT		
*C. fetus* subsp. *venerealis*	CTTAGCAGTTTGCGATATTGCCATT	142 bp	Hum et al. (1997)
	GCTTTTGAGATAACAATAAGAGCTT		
*C. jejuni*	CTATTTTATTTTTGAGTGCTTGTG	589 bp	Müller et al. (2011)
	GCTTTATTTGCCATTTGTTTTATTA		
*S.* Abortusovis	CGATGAAAGCGTAAATAAGG	900 bp	Beuzón et al. (1997)
	TTCTCTTGTCAGTCTCAAAC		
*Trueperella pyogenes*	GGCCCGAATGTCACCGC	270 bp	Ochi et al. (2020)
	AACTCCGCCTCTAGCGC		
*K. pneumoniae*	CAACGGTGTGGTTACTGACG	108 bp	Chander et al. (2011)
	TCTACGAAGTGGCCGTTTTC		

### 2.7 Statistical method

Vaginal swab PCR results were coded as binary outcomes (positive = 1, negative = 0). Culture results recorded as “antibiotic-resistant growth” or fungal contamination were considered indeterminate and excluded from the corresponding denominators. Between-group differences in pathogen prevalence were assessed using two-tailed Fisher's exact test. Because eight separate pathogen-specific comparisons were performed between repeat breeder and fertile control ewes, the Bonferroni correction was applied to control the familywise type I error rate associated with multiple hypothesis testing. Bonferroni-adjusted *p* values ( $p_{\mathrm{adj}}$ ) are reported throughout. Odds ratios (ORs) with 95 % confidence intervals (CIs) were estimated using the Haldane–Anscombe correction by adding 0.5 to each cell to accommodate zero-cell contingency tables. For each animal, the total number of PCR-detected pathogens was used as a co-infection burden score. Differences in this score among the three groups were evaluated using the Kruskal–Wallis test, followed by a post-hoc Mann–Whitney U test for pairwise comparison between Group 2 and Group 1. Wilson's score method was used to calculate 95 % CIs for individual proportions. All statistical analyses were performed using Python (version 3.12; Python Software Foundation, USA), with SciPy (version 1.13) and statsmodels (version 0.14).

## 3 Results

The ewes with infertility problems exhibited oestrus at regular intervals (17 ± 1 d). Estrous behaviour was characterised by surrounding the ram, following mating animals, and sniffing the scrotum of the ram. Of the 85 ewes that remained non-pregnant despite three consecutive matings, nine were biparous, whereas the remaining animals were multiparous ewes that had previously lambed three, four, or five times.

A total of 40 swab samples, including samples from 25 ewes and 15 rams, were analysed in the present study. Based on the culture results, 15 swab samples produced turbidity in *Mycoplasma* broth, indicating presumptive positivity. Following subculture on *Mycoplasma* agar, colonies exhibiting the characteristic “fried-egg” morphology of *Mycoplasma* spp. were observed in 12 samples. PCR analysis of DNA extracted from the *Mycoplasma* broth cultures detected *Mycoplasma* spp. in samples from 12 ewes and three rams.

Based on direct PCR analysis, *Campylobacter* spp. DNA was detected in swab samples from 10 ewes and five rams. However, all samples tested negative for *C. fetus* subsp. *fetus*, *C. fetus* subsp. *venerealis*, and *C. jejuni*. *Trueperella pyogenes* was detected in swab samples from 13 ewes and two rams. In addition, *Mycoplasma* spp. were detected in swab samples from 21 ewes and four rams, whereas *Mycoplasma bovigenitalium* was identified in 10 ewes and one ram. However, *M. canis*, *M. alkalescens*, *M. arginini*, *M. bovirhinis*, *M. agalactiae*, *M. putrefaciens*, *M. ovipneumoniae*, *Mycoplasma capricolum* subsp. *capripneumoniae*, *M. dispar*, and the *M. mycoides* cluster were not detected in any of the samples analysed. Likewise, DNA of *Brucella* spp., *Coxiella burnetii*, *Chlamydophila* spp., *Cp. abortus*, *Cp. pecorum*, *Salmonella* Abortusovis, and *Klebsiella pneumoniae* was not detected in any of the swab samples.

### 3.1 Pathogen prevalence by group

A total of 65 genital swab samples were examined by PCR, including 25 from Group 2 (repeat breeder ewes), 25 from Group 1 (clinically healthy ewes), and 15 from breeding rams used for natural mating. The proportions of PCR-positive animals for each of the eight target pathogens are presented in Table 2 and illustrated in Fig. 1.

**Table 2:** PCR detection rates and between-group comparison (Group 2 vs Group 1; Bonferroni-corrected Fisher's exact test).

Pathogen	Group 2 diseased	Rams	Group 1 healthy	OR	$p_{\mathrm{adj}}$
	( n=25 )	( n=15 )	( n=25 )	(95 % CI)	
*Mycoplasma* spp. (universal PCR)	21/25 (84.0 %)	4/15 (26.7 %)	0/25 (0.0 %)	∞ [12.4–4786]	< **0.001**
*M. bovigenitalium*	10/25 (40.0 %)	1/15 (6.7 %)	0/25 (0.0 %)	∞ [1.89–632]	**0.005**
*T. pyogenes*	13/25 (52.0 %)	2/15 (13.3 %)	6/25 (24.0 %)	3.43 [1.00–10.5]	0.634
*Campylobacter* spp.	10/25 (40.0 %)	5/15 (33.3 %)	8/25 (32.0 %)	1.42 [0.45–4.33]	1.000
*Staphylococcus* spp.	4/25 (16.0 %)	2/15 (13.3 %)	9/25 (36.0 %)	0.34 [0.10–1.32]	1.000
*Streptococcus* spp.	2/25 (8.0 %)	1/15 (6.7 %)	5/25 (20.0 %)	0.35 [0.08–1.98]	1.000
*Salmonella* spp.	1/25 (4.0 %)	1/15 (6.7 %)	6/25 (24.0 %)	0.13 [0.03–1.19]	0.786
*E. coli*	4/25 (16.0 %)	1/15 (6.7 %)	10/25 (40.0 %)	0.29 [0.09–1.11]	0.909

OR = odds ratio (Group 2 as numerator). ∞ indicates zero-cell table; OR estimated by Haldane correction. Bold font indicates statistically significant differences.

**Figure 1:**
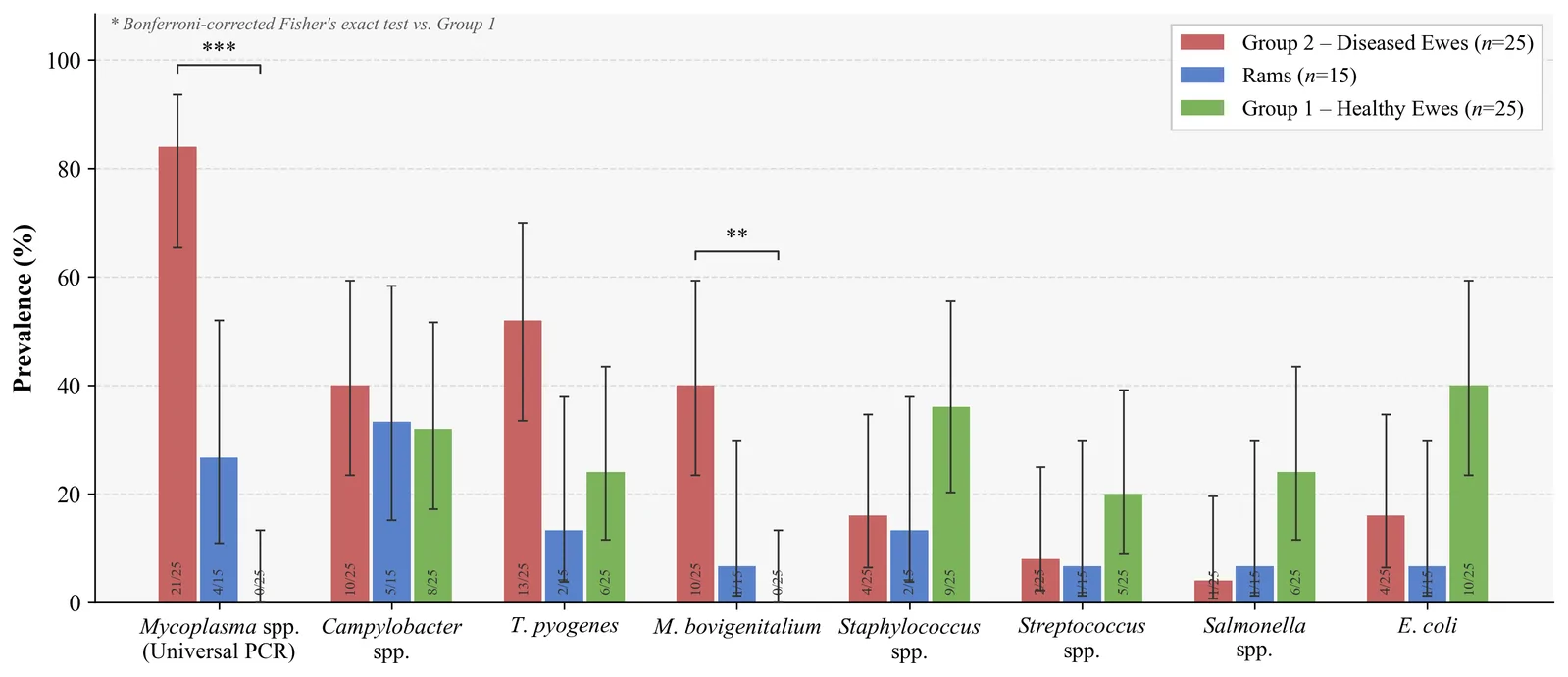
PCR-based prevalence of reproductive pathogens across the three study groups. Grouped bar chart showing the proportion (%) of PCR-positive animals for each of eight target pathogens in Group 2 – diseased ewes (red, n=25 ), rams (blue, n=15 ), and Group 1 – healthy ewes (green, n=25 ). Error bars represent 95 % Wilson score confidence intervals. Numbers inside each bar indicate the absolute count of positive animals over the total number tested for that pathogen (positive/n). Bracketed significance annotations above bars reflect Bonferroni-corrected two-tailed Fisher's exact tests comparing Group 2 vs Group 1: ** $p_{\mathrm{adj}}$ < 0.01; *** $p_{\mathrm{adj}}$ < 0.001; ns: not significant. *M. bovi.* = *Mycoplasma bovigenitalium*; *T. pyo.* = *Trueperella pyogenes*.

### 3.2 Detection in rams

Among the 15 rams, *Campylobacter* spp. was detected most frequently ( 5/15 ; 33.3 %), followed by *Mycoplasma* spp. ( 4/15 ; 26.7 %), *T. pyogenes* ( 2/15 ; 13.3 %), and *Staphylococcus* spp. ( 2/15 ; 13.3 %). The detection of *Mycoplasma* spp. in more than one-quarter of the breeding rams is epidemiologically noteworthy. Although the difference in *Mycoplasma* spp. prevalence between rams and healthy ewes did not survive Bonferroni correction ( $p_{\mathrm{adj}}$ = 0.12), the magnitude of positivity (26.7 %) and the biological plausibility of venereal transmission suggest that rams may serve as a reservoir and transmission vector for *Mycoplasma* spp. in this flock. Individual heatmap profiles (Fig. 2) confirm that several rams were positive for the same pathogen combinations identified in diseased ewes.

**Figure 2:**
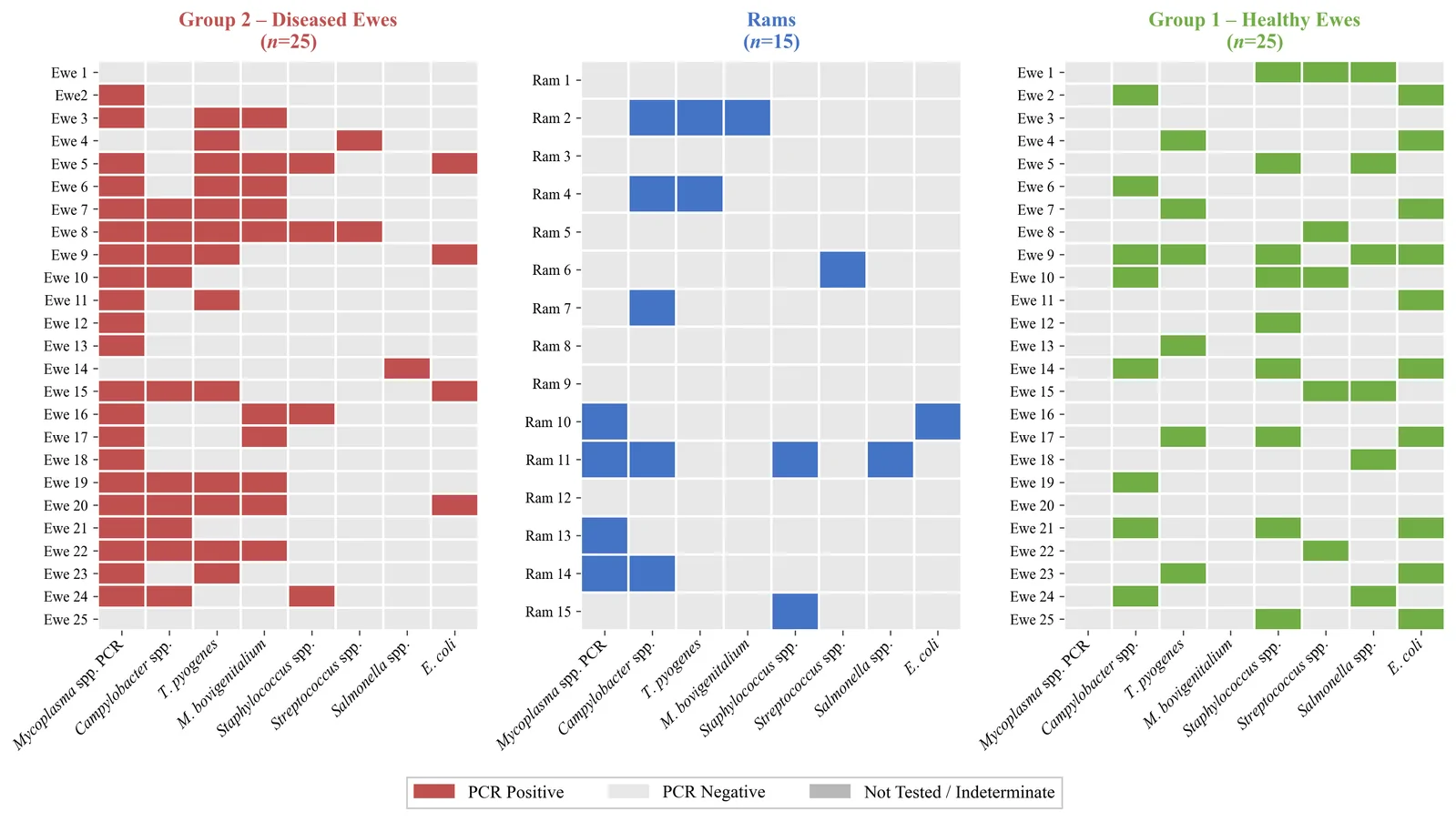
Individual-level PCR positivity profiles for all study animals by group and pathogen. Each row represents one animal; each column represents one of the eight target pathogens. Coloured cells indicate a PCR-positive result (red = Group 2 diseased ewes; blue = rams; green = Group 1 healthy ewes); light-grey cells indicate a PCR-negative result; medium-grey cells indicate samples that were not tested or yielded an indeterminate result (culture contaminated with antibiotic-resistant organisms or fungi). The heatmap illustrates the pattern of co-infections and pathogen co-occurrence within and between groups.

In summary, the findings provide strong statistical evidence that *Mycoplasma* spp., particularly *M. bovigenitalium*, is the pathogen most significantly associated with reproductive tract disease in ewes (Fig. 4). Direct PCR performed on vaginal swab samples is the most sensitive single diagnostic modality and should be considered the method of choice for field investigations of ovine vaginal discharge.

### 3.3 Organisms not significantly differing between groups

The prevalences of *Campylobacter* spp. (40.0 % vs. 32.0 %), *Staphylococcus* spp. (16.0 % vs 36.0 %), *Streptococcus* spp. (8.0 % vs 20.0 %), *Salmonella* spp. (4.0 % vs 24.0 %), and *E. coli* (16.0 % vs 40.0 %) did not differ significantly between Group 2 and Group 1 after correction (all $p_{\mathrm{adj}}$ > 0.78). Notably, *Staphylococcus* spp., *Salmonella* spp., and *E. coli* were numerically more prevalent in healthy ewes than in diseased ewes, consistent with their role as commensal members of the ovine genital tract microbiota rather than primary pathogens in this context.

### 3.4 Comparison of *Mycoplasma* diagnostic methods in diseased ewes

Among Group 2 ewes, direct swab-based universal PCR-detected *Mycoplasma* spp. in 21/25 animals (84.0 %), substantially outperforming both *Mycoplasma* broth culture ( 12/25 ; 48.0 %) and agar culture ( 12/25 ; 48.0 %). Of the 12 broth culture-positive samples subsequently subjected to PCR, all 12 (100 %) yielded a positive universal PCR result, and 11/12 (91.7 %) were confirmed as *M. bovigenitalium* by species-specific PCR (Fig. 3A). These findings indicate the near-complete dominance of *M. bovigenitalium* among culturable mycoplasma isolates from diseased ewes, whereas direct swab-based PCR identified an additional nine cases that were missed by culture alone. These data demonstrate that conventional culture underestimates the true prevalence of mycoplasmal infection in sheep with reproductive disease.

*Mycoplasma* broth cultures from nine of the 15 rams (60.0 %) were overgrown by antibiotic-resistant bacteria, rendering them non-evaluable and precluding culture-based assessment in the majority of the ram cohort. Direct PCR performed on ram swab samples was therefore the only reliable diagnostic modality for this group.

**Figure 3:**
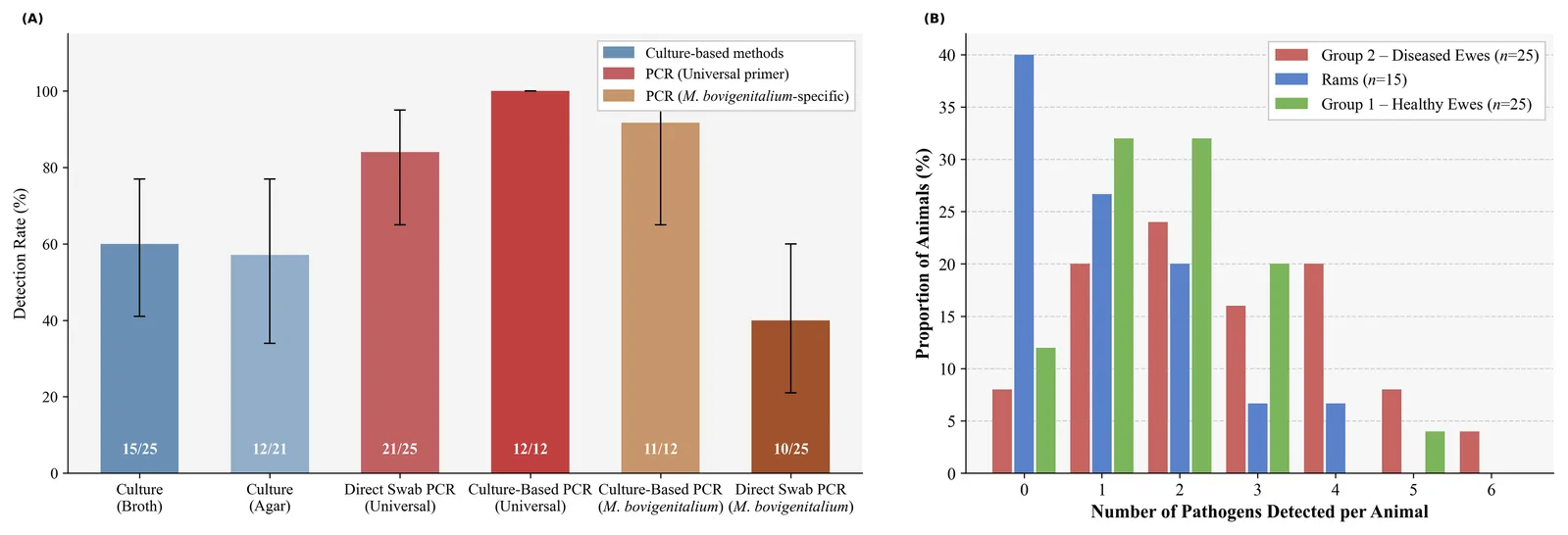
*Mycoplasma* diagnostic method comparison and co-infection burden distribution. **(A)** Detection rates for *Mycoplasma* spp. in Group 2 diseased ewes ( n=25 ) obtained by six different diagnostic approaches: direct culture in mycoplasma broth, direct culture on mycoplasma agar, direct swab-based universal PCR, culture broth-based universal PCR, culture broth-based *M. bovigenitalium*-specific PCR, and direct swab-based *M. bovigenitalium*-specific PCR. Numbers inside each bar indicate the number of positive results over the number of evaluable samples for that method. Error bars represent 95 % Wilson score confidence intervals. Culture-based PCR was performed only on broth culture-positive samples ( n=12 ); the denominator therefore differs from that of direct swab-based methods ( n=25 ). **(B)** Distribution of co-infection burden – defined as the total number of pathogens detected per animal by PCR – across the three study groups. Each cluster of bars represents a given number of simultaneously detected pathogens (0 through 6). Bar height indicates the proportion (%) of animals within each group carrying that number of pathogens. Group differences were assessed by the Kruskal–Wallis test ( H=9.52 , p=0.009 ).

### 3.5 Co-infection burden

The mean number of PCR-detected pathogens per animal differed significantly among the three groups (Kruskal–Wallis H=9.52 , p=0.009 ; Fig. 3B). Group 2 ewes harboured the highest co-infection burden (mean ± SD: 2.60 ± 1.61; range 0–6), followed by Group 1 healthy ewes (1.76 ± 1.16; range 0–5) and rams (1.13 ± 1.25; range 0–4). The pairwise difference between Group 2 and Group 1 approached, but did not reach, statistical significance (Mann–Whitney U=408 , p=0.059 ), likely reflecting the broad variability within both groups. Seven (28.0 %) diseased ewes were co-infected with three or more pathogens simultaneously, compared with three (12.0 %) healthy ewes.

### 3.6 Pathogens significantly associated with reproductive disease

*Mycoplasma* spp. was the pathogen most strongly associated with vaginal discharge. It was detected by universal PCR in 21 of 25 (84.0 %) diseased ewes (Group 2), whereas none of the 25 healthy ewes (Group 1) tested positive ( $p_{\mathrm{adj}}$ < 0.001; Fig. 4). *M. bovigenitalium* was identified in 10/25 (40.0 %) diseased ewes and, likewise, was not detected in any of the healthy ewes ( $p_{\mathrm{adj}}$ = 0.005). Among the rams, *Mycoplasma* spp. was detected by PCR in 4/15 (26.7 %) animals, of which one (6.7 %) tested positive specifically for *M. bovigenitalium*.

*T. pyogenes* was detected in 13/25 (52.0 %) Group 2 ewes compared with 6/25 (24.0 %) Group 1 ewes (OR = 3.43, 95 % CI 1.00–10.5; $p_{\mathrm{adj}}$ = 0.634; Fig. 4). Although this difference did not reach statistical significance after Bonferroni correction, reflecting the limited statistical power associated with the sample size, the direction and effect size were consistent with a pathological role for this organism in the reproductive tract of diseased animals.

**Figure 4:**
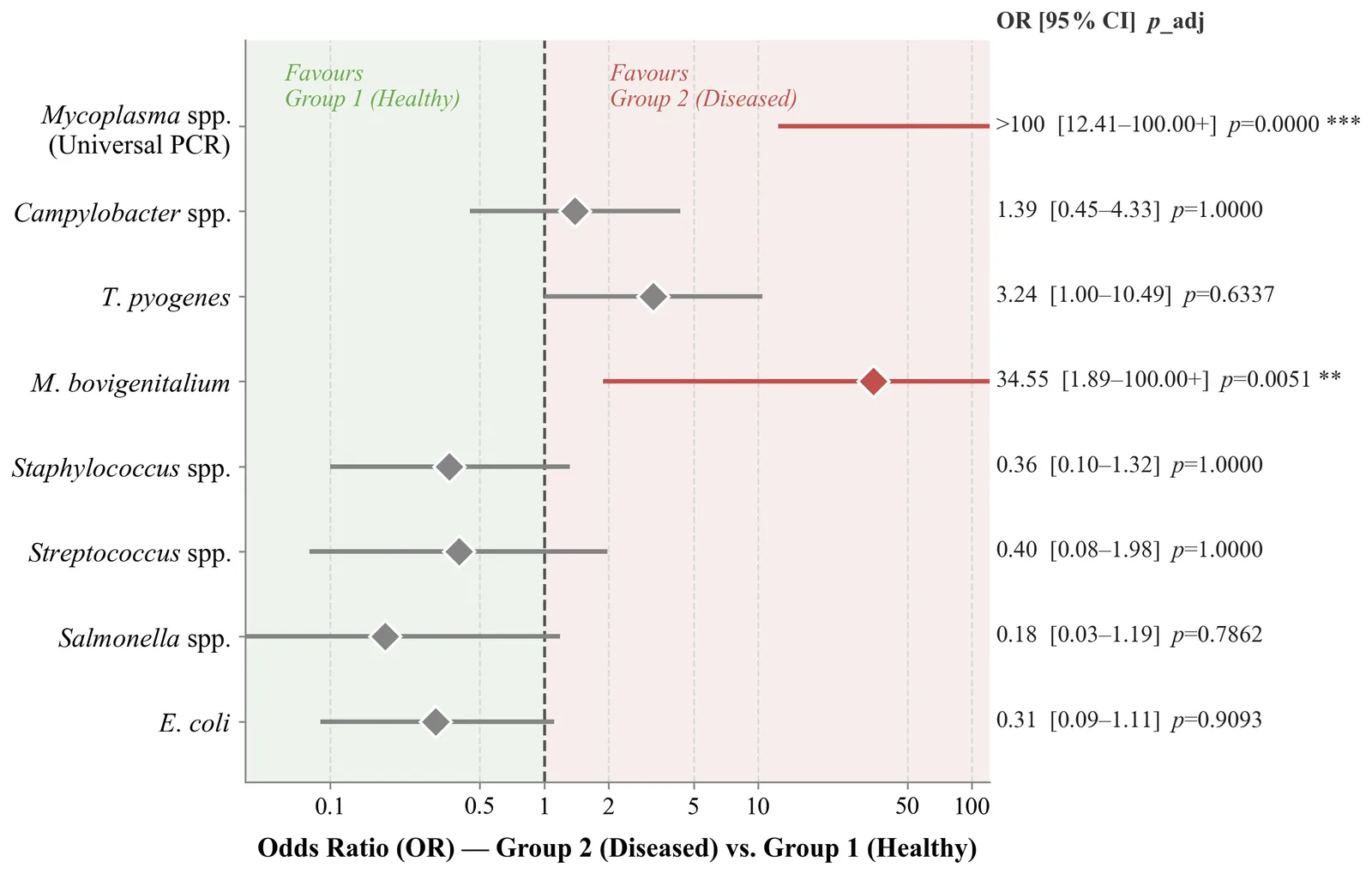
Forest plot of odds ratios for pathogen detection in Group 2 (diseased) versus Group 1 (healthy) ewes. Each row corresponds to one target pathogen. Filled diamonds indicate the point estimate of the odds ratio (OR); horizontal lines extend to the lower and upper bounds of the 95 % confidence interval. ORs were estimated using the Haldane–Anscombe correction (addition of 0.5 to each cell) to accommodate zero-cell contingency tables; upper CI bounds exceeding 100 are displayed as “ > 100”, and the plotted line is truncated at the axis limit for visual clarity. The dashed vertical line at OR = 1 represents the null hypothesis of no difference between groups. The red-shaded region (OR > 1) indicates a higher odds of detection in Group 2 (diseased), and the green-shaded region (OR < 1) indicates a higher odds in Group 1 (healthy). All p values are Bonferroni-corrected across the eight simultaneous comparisons: ** $p_{\mathrm{adj}}$ < 0.01; *** $p_{\mathrm{adj}}$ < 0.001. The x axis is displayed on a log scale.

## 4 Discussion

In this naturally managed sheep flock, repeat breeding was characterised by a distinct microbial signature dominated by *Mycoplasma* spp., particularly *M. bovigenitalium*, rather than by the classical abortifacient or systemic pathogens. In cattle, repeat breeding has long been regarded as a multifactorial condition involving nutritional, endocrine, managerial, and infectious influences, with subclinical endometritis frequently recognised as a common underlying pathway (Cummins et al., 2012; Salasel et al., 2010; Sood et al., 2015; Talukdar et al., 2016). In the present population, however, the findings support a predominantly infectious framework. This interpretation is strengthened by the study design, in which all ewes were managed under identical conditions throughout the breeding season, including continuous exposure to breeding rams, a common nutritional regimen, and a shared environment. By minimising variation attributable to non-infectious factors, this approach allowed the microbial component to be evaluated with minimal confounding. Moreover, because subclinical endometritis is rarely detectable during routine clinical examination and requires uterine cytology for definitive diagnosis (Salasel et al., 2010), a microbiological investigation was considered essential in the present study.

The association between parity and reproductive failure reported in cattle (Pérez-Marín and Quintela, 2023) was also observed in the present flock, where 76 of the 85 non-pregnant ewes were multiparous, having undergone three to five previous parturitions. This pattern suggests that cumulative uterine exposure to parturition-associated microbial challenges may progressively compromise endometrial defence mechanisms, a hypothesis that warrants further investigation in future longitudinal studies.

Before the microbial comparisons could be interpreted, the contribution of systemic infectious diseases had to be excluded. The detection of *C. burnetii* antibodies and BDV antigen in 21 ewes with reproductive failure – but without overt abortion – is consistent with the well-recognised subclinical presentation of both pathogens in sheep. *C. burnetii* has a more pronounced reproductive impact in goats than in sheep (Dijkstra et al., 2012; Rousset et al., 2009); and its subtle, often undetected persistence across consecutive lambing seasons represents an underestimated management risk (Álvarez-Alonso et al., 2018). BDV, with a reported seroprevalence of 5 %–50 % in affected flocks (Monies et al., 2004; Nettleton et al., 1998), was detected in approximately 2.3 % of animals in the present flock. Its co-occurrence with *C. burnetii* seropositivity is consistent with previous reports suggesting that co-infection may synergistically contribute to reproductive failure (Feknous et al., 2018). Excluding these co-infected animals before microbiological analysis ensured that the subsequent comparisons between Group 1 and Group 2 reflected differences attributable to genital tract pathogens rather than to systemic infectious diseases.

With these animals excluded, the remaining microbial profile distinguished pathogens that discriminated between groups from those that did not. *Salmonella* Abortusovis was not detected in any vaginal or preputial swab sample, and other *Salmonella* spp. were present at comparable prevalences across the groups, supporting a commensal rather than pathogenic role for this genus in the present context. *Chlamydia* spp. were likewise absent from all samples, despite their well-documented association with urogenital infections and reduced conception rates in small ruminants (Bowen et al., 1978; Shewen, 1980; Wilsmore et al., 1990), suggesting that chlamydial infection did not contribute to reproductive failure in this flock. These negative findings are informative in their own right. Against a background in which *Salmonella*, *Chlamydia*, *Staphylococcus*, *Streptococcus*, and *E. coli* showed no differential distribution between the groups, the marked increase in *Mycoplasma* spp. and *M. bovigenitalium* among diseased ewes emerges as a distinct biological signal rather than the result of non-specific contamination.

### 4.1 *Mycoplasma* spp. as the dominant pathogen in diseased ewes

The 84.0 % detection rate of *Mycoplasma* spp. in Group 2 ewes exceeds the 30 %–60 % prevalence reported in earlier culture-based studies of ewes with vulvovaginitis or vaginal discharge (Cottew, 1984; Nicholas et al., 2008). This discrepancy is most likely attributable to the diagnostic limitations of conventional culture rather than to a true difference in pathogen burden, as direct-swab PCR identified nine additional positive cases that were not detected by culture. *Mycoplasma bovigenitalium* was first isolated from the ovine genital tract in association with mild vulvovaginitis and endometritis by Jones (Jones et al., 1985), and subsequent molecular surveys have identified it as one of the most prevalent mycoplasmal pathogens of the sheep reproductive tract across geographically diverse populations (Halium et al., 2019; Stipkovits and Kempf, 1996). Its complete absence from all 25 healthy control ewes, yielding an undefined odds ratio, argues against a commensal or incidental carrier status and supports a pathogenic role in ovine reproductive tract disease.

Two features distinguish the present findings from those of previous descriptive surveys. First, the pathogen was detected in both ewes and rams within a single infertile flock. Second, it consistently co-occurred with *Trueperella pyogenes* in the diseased phenotype. Mycoplasmal species have been reported to be entirely absent from microbiome profiles obtained throughout healthy ovine pregnancies (Cassas et al., 2024), reinforcing the interpretation that their detection in non-pregnant ewes reflects a pathological rather than a normal colonisation state. Collectively, these observations provide, to the best of our knowledge, the first systematic characterisation of *M. bovigenitalium* as a contributor to reproductive failure in a naturally managed sheep flock under field conditions.

The reservoir for this pathogen appears to extend to rams. *Mycoplasma* spp. were detected in 26.7 % of rams, with one-quarter of the positive animals confirmed as *M. bovigenitalium*, consistent with the well-established ability of rams to harbour mycoplasmas within the prepuce and urogenital tract as long-term asymptomatic carriers (DaMassa et al., 1992; Nicholas, 2002). The concordance between the pathogen profiles of rams and ewes (Fig. 2), together with the exclusive use of natural mating in this flock, suggests that venereal transmission is a plausible route of infection. This interpretation is further supported by an Australian study involving 629 cattle, in which mycoplasmal colonisation, predominantly *M. bovigenitalium*, increased following natural mating, consistent with venereal acquisition (Hazelton et al., 2020). The same transmission dynamic is biologically plausible in sheep and warrants further investigation in prospective studies.

### 4.2 *Trueperella pyogenes* and polymicrobial interactions

The co-occurrence of *T. pyogenes* and *M. bovigenitalium* emerged as a consistent feature of the diseased phenotype. *T. pyogenes* (formerly *Arcanobacterium pyogenes*) was detected in 52.0 % of diseased ewes compared with 24.0 % of healthy ewes (OR = 3.43, 95 % CI 1.00–10.5). It is a well-characterised opportunistic pathogen of the ruminant reproductive tract and is routinely isolated from cases of metritis, endometritis, and pyometra in cattle, sheep, and goats, where it typically acts synergistically with other bacteria following primary mucosal disruption (Jost and Billington, 2005; Rzewuska et al., 2019). In cattle, its synergistic interaction with *M. bovigenitalium* following a complicated puerperium has been shown to sustain subclinical endometritis (Osawa, 2021). The present study identified a comparable pattern in sheep, with the two organisms co-detected in the vaginal swabs of most infertile ewes and jointly isolated from the preputial swab of one ram. To the best of our knowledge, this co-infection pattern has not previously been described in ovine populations. The moderate prevalence of *T. pyogenes* in healthy ewes (24.0 %) is consistent with commensal carriage within the normal genital microbiota, with pathogenicity likely emerging under conditions of immunosuppression or concurrent mycoplasmal infection that compromises mucosal barrier integrity, a model supported by the co-infection data presented in the present study.

In contrast, *Campylobacter* spp., *Staphylococcus* spp., *Streptococcus* spp., *Salmonella* spp., and *Escherichia coli* showed no significant differences in prevalence between the groups, suggesting that these organisms were not associated with repeat breeding in the present flock. *Campylobacter* spp. were detected in approximately one-third of animals across all groups, a prevalence comparable to that reported in apparently healthy sheep populations. Although certain species, particularly *C. fetus* subsp. *fetus*, remain important causes of ovine abortion under specific flock conditions (Mshelia et al., 2010; Sahin et al., 2017), species-specific PCR did not detect *C. fetus* subsp. *fetus*, *C. fetus* subsp. *venerealis*, or *C. jejuni* in the present study. These findings suggest that the *Campylobacter* spp. detected were unlikely to have contributed to the reproductive failure observed, although broader species-level characterisation would be valuable in future investigations.

### 4.3 Statistical interpretation of the marginal associations

Two of the principal associations – the difference in *Mycoplasma* spp. prevalence between rams and ewes ( $p_{\mathrm{adj}}$ = 0.12) and the increased prevalence of *T. pyogenes* in diseased ewes ( $p_{\mathrm{adj}}$ = 0.634) – did not remain statistically significant after Bonferroni correction. This outcome most likely reflects the conservative nature of the correction in combination with the moderate sample size, both of which reduce the statistical power to detect effects of the observed magnitude, rather than indicating a lack of biological relevance. The effect size for *T. pyogenes* (OR ≈ 3.4) is comparable to associations considered aetiologically relevant in the bovine endometritis literature, while the concordance between rams and ewes is further supported by the observed co-infection patterns and the proposed venereal transmission framework described above. These associations should therefore be regarded as biologically and clinically meaningful findings that warrant confirmation in adequately powered cohorts and are interpreted throughout the present study accordingly, rather than as definitive evidence of causality.

### 4.4 Diagnostic superiority of direct PCR and co-infection burden

The 36 percentage-point difference between the PCR (84.0 %) and culture (48.0 %) detection rates for *Mycoplasma* spp. represents the most clinically relevant diagnostic finding of the present study. Mycoplasmas present inherent challenges for culture, including fastidious growth requirements, prolonged generation times, and susceptibility to overgrowth by contaminating flora, all of which render conventional culture unreliable under field conditions. This limitation was reflected in the present study by the 60 % non-evaluable rate among ram cultures due to overgrowth by antibiotic-resistant bacteria. This pattern is consistent with findings in the bovine and caprine literature, where PCR-based methods have repeatedly demonstrated superior sensitivity compared with culture (Nicholas and Ayling, 2003; Wisselink et al., 2019). Species-specific PCR confirmed *M. bovigenitalium* in 91.7 % of the culture-confirmed broth samples, establishing this species as the predominant culturable mycoplasmal isolate in clinically affected ewes and a clear diagnostic target for future flock surveillance. These findings indicate that direct-swab PCR should replace culture as the first-line diagnostic method for field investigations of ovine vaginal discharge and that negative culture results should not be used to exclude a mycoplasmal aetiology.

Beyond detection sensitivity, the co-infection burden itself distinguished the diseased animals. Diseased ewes harboured a higher mean pathogen load than healthy ewes (2.60 vs 1.76 pathogens per animal; Kruskal–Wallis, p=0.009 ), and 28 % carried three or more pathogens simultaneously, more than twice the proportion observed in the healthy controls. These findings suggest that the most severe clinical presentations are characterised by polymicrobial rather than single-pathogen infections, consistent with a cascade model in which primary mycoplasmal infection compromises mucosal immune defences and facilitates secondary colonisation by opportunistic organisms such as *T. pyogenes* and *E. coli* – analogous to the mechanisms described in bovine metritis (Sheldon et al., 2009). Clinically, this profile suggests that antimicrobial regimens directed against a single pathogen are unlikely to be sufficient; instead, combination treatment strategies targeting both the mycoplasmal and opportunistic bacterial components should be evaluated in future studies.

### 4.5 Limitations and future directions

Several limitations should be considered when interpreting these findings. First, the cross-sectional study design precludes temporal inference regarding the sequence of pathogen acquisition and the onset of clinical disease and does not permit definitive conclusions about causality. Longitudinal studies incorporating pre-mating and serial post-mating sampling are therefore required to determine whether mycoplasmal colonisation precedes reproductive failure or develops as a secondary consequence of impaired uterine immunity. Second, the single-flock design limits the generalisability of the prevalence estimates to other geographic regions and production systems. Third, the absence of sequencing-based strain characterisation or multilocus sequence typing (MLST) limited our ability to provide direct molecular evidence of venereal transmission between rams and ewes, and these approaches should be prioritised in future investigations. Finally, the moderate sample size ( n=65 ) reduced statistical power, contributing to wide confidence intervals and preventing marginal associations from reaching statistical significance after correction. Larger cohorts combined with whole-metagenome sequencing of the reproductive tract microbiome throughout the mating cycle would substantially enhance our mechanistic understanding of host–pathogen interactions underlying ovine reproductive failure.

## 5 Conclusions

The present study advances our understanding of ovine reproductive failure by identifying *Mycoplasma* spp., particularly *M. bovigenitalium*, as the pathogen most significantly and consistently associated with reproductive tract disease in ewes, while demonstrating that breeding rams may serve as asymptomatic venereal reservoirs within affected flocks. The concurrent detection of *M. bovigenitalium* and *Trueperella pyogenes* as a characteristic co-infection pattern of the diseased phenotype, together with the near-complete absence of both organisms in healthy controls, suggests a polymicrobial pathogenic model that has not previously been described in sheep and warrants prospective validation. Beyond its aetiological implications, the present study provides clear evidence that direct-swab PCR substantially outperforms conventional culture for the detection of mycoplasmas under field conditions and should be adopted as the first-line diagnostic method in flock investigations. Pre-mating PCR screening of breeding rams, combined with targeted treatment and the isolation of PCR-positive animals, represents the most evidence-based strategy currently available for reducing the burden of mycoplasma-associated reproductive failure in sheep flocks.

## Data Availability

The data underlying this study contain potentially identifying information about the participating commercial sheep farm. To protect the confidentiality of the farms, the full dataset is available from the corresponding author upon reasonable request and subject to appropriate data-sharing agreements.

## References

[bib1.bib1] Abecia JA, Forcada F, González-Bulnes A (2012). Hormonal control of reproduction in small ruminants. Anim Reprod Sci.

[bib1.bib2] Álvarez-Alonso R, Barandika JF, Ruiz-Fons F, Ortega-Araiztegi I, Jado I, Hurtado A, García-Pérez AL (2018). Stable levels of *Coxiella burnetii* prevalence in dairy sheep flocks but changes in genotype distribution after a 10-year period in northern Spain. Acta Vet Scand.

[bib1.bib3] Bashiruddin JB, Miles RJ, Nicholas RAJ (1998). Mycoplasma Protocols, Methods in Molecular Biology.

[bib1.bib4] Berri M, Laroucau K, Rodolakis A (2000). The detection of *Coxiella burnetii* from ovine genital swabs, milk and fecal samples by the use of a single touchdown polymerase chain reaction. Vet Microbiol.

[bib1.bib5] Berri M, Rekiki A, Boumedine KS, Rodolakis A (2009). Simultaneous differential detection of *Chlamydophila abortus, *Chlamydophila pecorum* and *Coxiella burnetii* from aborted ruminant's clinical samples using multiplex PCR*. BMC Microbiol.

[bib1.bib6] Beuzón CR, Schiaffino A, Leori G, Cappuccinelli P, Rubino S, Casadesús J (1997). Identification of Salmonella abortusovis by PCR amplification of a serovar-specific IS200 element. Appl Environ Microb.

[bib1.bib7] Bowen RA, Spears P, Storz J, Seidel Jr. GE (1978). Mechanisms of infertility in genital tract infections due to Chlamydia psittaci transmitted through contaminated semen. J Infect Dis.

[bib1.bib8] Cassas MS, Jonas LC, Anderson CJ, Schmitz-Esser S, Youngs CR (2024). Temporal changes in ewe vaginal microbiota throughout gestation. Front Microbiol.

[bib1.bib9] Chander Y, Ramakrishnan MA, Jindal N, Hanson K, Goyal SM (2011). Differentiation of *Klebsiella pneumoniae* and *K. oxytoca* by multiplex polymerase chain reaction. International Journal of Applied Research in Veterinary Medicine.

[bib1.bib10] Cottew GS (1984). Overview of mycoplasmoses in sheep and goats. Israel J Med Sci.

[bib1.bib11] Cummins SB, Lonergan P, Evans ACO, Butler ST (2012). Genetic merit for fertility traits in Holstein cows: II. Ovarian follicular and corpus luteum dynamics, reproductive hormones, and estrus behavior. J Dairy Sci.

[bib1.bib12] DaMassa AJ, Wakenell PS, Brooks DL (1992). Mycoplasmas of goats and sheep. J Vet Diagn Invest.

[bib1.bib13] Dijkstra F, van der Hoek W, Wijers N, Schimmer B, Rietveld A, Wijkmans CJ, Vellema P, Schneeberger PM (2012). The 2007–2010 Q fever epidemic in The Netherlands: characteristics of notified acute Q fever patients and the association with dairy goat farming. FEMS Immunol Med Mic.

[bib1.bib14] Feknous N, Hanon J-B, Tignon M, Khaled H, Bouyoucef A, Cay B (2018). Seroprevalence of border disease virus and other pestiviruses in sheep in Algeria and associated risk factors. BMC Vet Res.

[bib1.bib15] Givens MD, Marley MSD (2008). Infectious causes of embryonic and fetal mortality. Theriogenology.

[bib1.bib16] Halium MMA, Salib FA, Marouf SA, Massieh ESA (2019). Isolation and molecular characterization of *Mycoplasma* spp. in sheep and goats in Egypt. Veterinary World.

[bib1.bib17] Hazelton MS, Morton JM, Bosward KL, Sheehy PA, Parker AM, Dwyer CJ, Niven PG, House JK (2020). *Mycoplasma* species in vaginas of dairy cows before and after exposure to bulls and their association with conception. J Dairy Sci.

[bib1.bib18] Hum S, Quinn K, Brunner J, On SLW (1997). Evaluation of a PCR assay for identification and differentiation of *Campylobacter fetus* subspecies. Aust Vet J.

[bib1.bib19] Jones GE, Keir WA, Gilmour JS (1985). The pathogenicity of *Mycoplasma ovipneumoniae* and *Mycoplasma arginini* in ovine and caprine tracheal organ cultures. J Comp Pathol.

[bib1.bib20] Jost BH, Billington SJ (2005). *Arcanobacterium pyogenes*: molecular pathogenesis of an animal opportunist. Antonie van Leeuwenhoek.

[bib1.bib21] Kemmerling K, Müller U, Mielenz M, Sauerwein H (2009). *Chlamydophila* species in dairy farms: polymerase chain reaction prevalence, disease association, and risk factors identified in a cross-sectional study in western Germany. J Dairy Sci.

[bib1.bib22] Kennedy D (2012). Understanding Lamb Mortality, Ontario Ministry of Agriculture, Food and Rural Affairs.

[bib1.bib23] Kobayashi H, Hirose K, Worarach A, Paugtes P, Ito N, Morozumi T, Yamamoto K (1998). In vitro amplification of the 16S rRNA genes from *Mycoplasma bovirhinis*, *Mycoplasma alkalescens* and *Mycoplasma bovigenitalium* by PCR. J Vet Med Sci.

[bib1.bib24] Krupová Z, Krupa E, Wolfová M, Michaličková M (2014). Impact of variation in production traits, inputs costs and product prices on profitability in multi-purpose sheep. Span J Agric Res.

[bib1.bib25] Lee PY, Costumbrado J, Hsu CY, Kim YH (2012). Agarose gel electrophoresis for the separation of DNA fragments. J Vis Exp.

[bib1.bib26] Le Grand D, Saras E, Blond D, Solsona M, Poumarat F (2004). Assessment of PCR for routine identification of species of the *Mycoplasma mycoides* cluster in ruminants. Vet Res.

[bib1.bib27] Marques LM, Buzinhani M, Yamaguti M, Oliveira RC, Ferreira JB, Mettifogo E, Timenetsky J (2007). Use of a polymerase chain reaction for detection of *Mycoplasma dispar* in the nasal mucus of calves. J Vet Diagn Invest.

[bib1.bib28] McAuliffe L, Ellis RJ, Ayling RD, Nicholas RAJ (2003). Differentiation of *Mycoplasma* species by 16S ribosomal DNA PCR and denaturing gradient gel electrophoresis fingerprinting. J Clin Microbiol.

[bib1.bib29] Menzies PI, Youngquist RS, Threlfall WR (2007). Current Therapy in Large Animal Theriogenology.

[bib1.bib30] Menzies PI (2011). Control of important causes of infectious abortion in sheep and goats. Vet Clin N Am-Food A.

[bib1.bib31] Mobini S, Youngquist RS, Threlfall WR (2007). Infectious causes of abortion. Current Therapy in Large Animal Theriogenology.

[bib1.bib32] Monies RJ, Paton DJ, Vilcek S (2004). Mucosal disease-like lesions in sheep infected with Border disease virus. Vet Rec.

[bib1.bib33] Mshelia GD, Amin JD, Woldehiwet Z, Murray RD, Egwu GO (2010). Epidemiology of bovine venereal campylobacteriosis: geographic distribution and recent advances in molecular diagnostic techniques. Reprod Domest Anim.

[bib1.bib34] Müller W, Böhland C, Methner U (2011). Detection and genotypic differentiation of *Campylobacter jejuni* and *Campylobacter coli* strains from laying hens by multiplex PCR and *fla*-typing. Res Vet Sci.

[bib1.bib35] Nettleton PF, Gilray JA, Russo P, Dlissi E (1998). Border disease of sheep and goats. Vet Res.

[bib1.bib36] Nicholas RAJ (2002). Improvements in the diagnosis and control of diseases of small ruminants caused by mycoplasmas. Small Ruminant Res.

[bib1.bib37] Nicholas RAJ, Ayling RD (2003). *Mycoplasma bovis*: disease, diagnosis, and control. Res Vet Sci.

[bib1.bib38] Nicholas RAJ, Baker SE, Miles RJ, Nicholas RAJ (1998). Recovery of mycoplasmas from animals. Mycoplasma Protocols, Methods in Molecular Biology.

[bib1.bib39] Nicholas RAJ, Ayling RD, McAuliffe L (2008). Mycoplasma diseases of ruminants.

[bib1.bib40] Ochi K, Okamoto M, Okamoto M, Okura M, Takamatsu D (2020). Development of a novel *Trueperella pyogenes*-specific PCR assay. J Vet Med Sci.

[bib1.bib41] Osawa T (2021). Predisposing factors, diagnostic and therapeutic aspects of persistent endometritis in postpartum cows. J Reprod Develop.

[bib1.bib42] Pérez-Marín CC, Quintela LA (2023). Current insights in the repeat breeder cow syndrome. Animals.

[bib1.bib43] Ratanapob N, VanLeeuwen J, McKenna S, Wichtel M, Stryhn H, Rodriguez-Lecompte JC, Menzies P, Wichtel J (2020). Management factors associated with perinatal lamb mortality in Prince Edward Island flocks. Prev Vet Med.

[bib1.bib44] Reinoso-Peláez EL, Saura M, González C, Ramón M, Calvo JH, Serrano M (2025). The influence of vaginal microbiota on ewe fertility: a metagenomic and functional genomic approach. Microbiome.

[bib1.bib45] Romero C, Gamazo C, Pardo M, López-Goñi I (1995). Specific detection of Brucella DNA by PCR. J Clin Microbiol.

[bib1.bib46] Rousset E, Durand B, Champion JL, Prigent M, Dufour P, Forfait C, Marois M, Gasnier T, Duquesne V, Thiéry R (2009). Efficiency of a phase 1 vaccine for the reduction of vaginal *Coxiella burnetii* shedding in a clinically affected goat herd. Clin Microbiol Infec.

[bib1.bib47] Rzewuska M, Kwiecień E, Chrobak-Chmiel D, Kizerwetter-Świda M, Stefańska I, Gieryńska M (2019). Pathogenicity and virulence of *Trueperella pyogenes*: a review. Int J Mol Sci.

[bib1.bib48] Sahin O, Yaeger M, Wu Z, Zhang Q (2017). *Campylobacter*-associated diseases in animals. Annu Rev Anim Biosci.

[bib1.bib49] Salasel B, Mokhtari A, Taktaz T (2010). Prevalence, risk factors for and impact of subclinical endometritis in repeat breeder dairy cows. Theriogenology.

[bib1.bib50] Shah BR (2019). Factors leading to early embryonic death. Nepalese Veterinary Journal.

[bib1.bib51] Sheldon IM, Cronin J, Goetze L, Donofrio G, Schuberth HJ (2009). Defining postpartum uterine disease and the mechanisms of infection and immunity in the female reproductive tract in cattle. Biol Reprod.

[bib1.bib52] Shewen PE (1980). Chlamydial infection in animals: a review. Canadian Vet J.

[bib1.bib53] Sood P, Zachut M, Dube H, Moallem U (2015). Behavioral and hormonal pattern of repeat breeder cows around estrus. Reproduction.

[bib1.bib54] Stipkovits L, Kempf I (1996). Mycoplasmoses in poultry. Revue Scientifique et Technique (International Office of Epizootics).

[bib1.bib55] Subramaniam S, Bergonier D, Poumarat F, Capaul S, Schlatter Y, Nicolet J, Frey J (1998). Species identification of *Mycoplasma bovis* and *Mycoplasma agalactiae* based on the *uvrC* genes by PCR. Mol Cell Probe.

[bib1.bib56] Talukdar DJ, Talukdar P, Ahmed K (2016). Minerals and its impact on fertility of livestock: a review. Agricultural Reviews.

[bib1.bib57] Timenetsky J, Santos LM, Buzinhani M, Mettifogo E (2006). Detection of multiple mycoplasma infection in cell cultures by PCR. Braz J Med Biol Res.

[bib1.bib58] Vanniasinkam T, Lanser JA, Barton MD (1999). PCR for the detection of *Campylobacter* spp. in clinical specimens. Lett Appl Microbiol.

[bib1.bib59] Wilsmore AJ, Izzard KA, Wilsmore BC, Dagnall GJR (1990). Breeding performance of sheep infected with Chlamydia psittaci (ovis) during their preceding pregnancy. Vet Rec.

[bib1.bib60] Wisselink HJ, Smid B, Plater J, Ridley A, Andersson AM, Aspán A, Pohjanvirta T, Vähänikkilä N, Larsen H, Høgberg J, Colin A, Tardy F (2019). A European interlaboratory trial to evaluate the performance of different PCR methods for *Mycoplasma bovis* diagnosis. BMC Vet Res.

[bib1.bib61] WOAH (2018). Manual of Diagnostic Tests and Vaccines for Terrestrial Animals.

[bib1.bib62] Wong-Lee JG, Lovett M, Persing DH, Smith TF, Tenover FC, Smith TJ (1993). Diagnostic Molecular Microbiology: Principles and Applications.

[bib1.bib63] Woubit S, Lorenzon S, Peyraud A, Manso-Silván L, Thiaucourt F (2004). A specific PCR for the identification of *Mycoplasma capricolum* subsp. *capripneumoniae*, the causative agent of contagious caprine pleuropneumonia (CCPP). Vet Microbiol.

